# Editorial for the Special Issue “Aging, Age-Related Changes in the Brain and the Progression of Alzheimer’s Disease”

**DOI:** 10.3390/cimb47050318

**Published:** 2025-04-29

**Authors:** A. N. M. Mamun-Or-Rashid

**Affiliations:** 1 Anti-Aging Medical Research Center, Graduate School of Life and Medical Sciences, Doshisha University, 1-3 Tatara Miyakodani, Kyoto 610-0394, Japan; anm541@pitt.edu; 2 Glycation Stress Research Center, Graduate School of Life and Medical Sciences, Doshisha University, 1-3 Tatara Miyakodani, Kyoto 610-0394, Japan; 3 Department of Environmental and Occupational Health, University of Pittsburgh, Pittsburgh, PA 15261, USA

## 1. Alzheimer’s Disease and Aging

Alzheimer’s disease (AD) is a complex neurodegenerative disorder characterized by progressive cognitive decline, memory impairment, and synaptic dysfunction. Despite extensive research, the underlying mechanisms driving AD pathogenesis remain elusive. In this editorial, we discuss nine recently published manuscripts that shed light on diverse aspects of AD, ranging from potential drug candidates to microorganisms’ role, biomarker exploration, and the impact of insulin on microglial activation. The amalgamation of these findings offers a multifaceted approach to understanding AD and paves the way for innovative therapeutic strategies.

## 2. Novel Drug Candidates

One of the most promising avenues in AD research is the development of pharmacological interventions that target synaptic integrity. Zernov et al. introduced *N*-(2-chlorophenyl)-2-(4-phenylpiperazine-1-yl) acetamide (51164), a TRPC6-positive modulator that upregulates postsynaptic neuronal store-operated calcium entry, maintains mushroom spine density, and restores synaptic plasticity in amyloidogenic mouse models. Additionally, the study identified an alternative mechanism involving actin filament stabilization, highlighting a potential therapeutic target for slowing disease progression. These findings aligned with a later study on C20, a TRPC6 agonist shown to enhance synaptic plasticity and cognitive function through complementary mechanisms, supported by in silico, in vitro, ex vivo, pharmacokinetic, and in vivo studies [1].

## 3. Genetic Associations and Biomarker Exploration

Beyond pharmacological interventions, genetic and biomarker studies continue to refine our understanding of AD susceptibility. The study by Siokas et al. on the SOD2 rs4880 variant highlighted the complexities of genetic associations in AD. While no significant correlation was found in their cohort, conflicting findings from previous studies [2] emphasize the necessity for larger and more diverse genetic analyses. Such research is critical in identifying genetic risk factors and potential biomarker candidates that can aid in early diagnosis.

## 4. Impact of Sepsis on AD Pathology

The study by Rotaru-Zavaleanu et al. demonstrated that sepsis may accelerate amyloid plaque formation and cognitive decline, reinforcing the link between systemic inflammation and neurodegeneration. Given that sepsis survivors face an increased risk of dementia, these findings highlight the need for targeted interventions to mitigate inflammatory responses in vulnerable populations. A study demonstrated that bacterial sepsis increased the hippocampal fibrillar amyloid plaque load and neuroinflammation in a mouse model of AD, indicating that sepsis can worsen amyloid deposition and related inflammation [3]. Furthermore, clinical observations have linked sepsis to long-term cognitive decline. A systematic review and meta-analysis found that sepsis survivors have an increased risk of all-cause dementia, emphasizing the importance of appropriate management and prevention strategies to preserve cognitive function in these individuals [4]. These studies collectively underscore the significance of systemic inflammation, such as that induced by sepsis, in influencing AD pathology and cognitive decline. They highlight the necessity for further research into the mechanisms by which systemic infections contribute to neurodegenerative processes and the development of interventions to mitigate these effects.

## 5. Amyloid Peptide as an RNA Recognition/Binding Peptide

The manuscript by Nahalka introduced a novel model for Alzheimer’s disease (AD) pathogenesis, proposing that the β-amyloid (Aβ) peptide functions as an RNA recognition and binding molecule. This hypothesis introduces a potential link between Aβ, RNA metabolism, and neurodegeneration, broadening the conventional amyloid hypothesis. The hypothesis was supported by bioinformatics analyses revealing sequences within Aβ compatible with RNA regulatory elements, suggesting potential roles in RNA processing, transport, and stability. This model offers a framework to explain various aspects of AD pathophysiology, including inflammation and neurodegeneration [5]. While the traditional amyloid hypothesis focuses on Aβ aggregation as a central event in AD, Nahalka’s model suggested a functional role for Aβ in RNA metabolism, potentially linking Aβ’s role to the observed dysregulation of RBPs and RNA dynamics in AD. This perspective encourages further investigation into the interactions between Aβ and RNA, which could lead to new therapeutic targets addressing the RNA-related aspects of AD pathology.

## 6. Mitochondrial DNA in AD Pathology

Mitochondrial dysfunction and DNA leakage into the cytoplasm have been implicated in AD pathology. Hussan et al. introduced a method to evaluate ectopic mitochondrial DNA (mtDNA) in cells, highlighting its potential relevance in understanding various diseases, including AD. The work emphasized the importance of quantifying mitochondrial DNA to investigate its role in neurodegenerative disorders and various other conditions [6]. As mtDNA is increasingly recognized as a trigger for microglial activation and neuroinflammatory cascades, understanding these processes may open new therapeutic avenues.

## 7. Nutritional Approaches in AD Management

A nutritional approach to AD management was reviewed by Palimariciuc et al. Based on AD’s multifactorial origin, nutrition may significantly contribute to the complex pathological picture. The manuscript identified potential dietary compounds that could modulate cognitive status and provide neuroprotection. By focusing on nutrients’ roles in AD pathophysiology, researchers aim to formulate personalized diets for improved AD management [6]. A systematic review published in *Frontiers in Neuroscience* highlighted that adherence to the Mediterranean diet is associated with improved cognitive outcomes, increased gray matter volume, and decreased memory decline [7]. These studies collectively underscore the potential of tailored nutritional approaches in improving cognitive function and overall well-being in individuals with Alzheimer’s disease.

## 8. Insulin’s Role in Microglial Activation

Insulin’s involvement in AD pathogenesis is a subject of interest. You et al. reviewed insulin’s pivotal role in learning and memory and its regulation of tau phosphorylation through the PI3K-Akt-GSK3β signaling pathway. Additionally, they explored insulin’s potential impact on microglia activation in AD pathology. The review calls for further investigations to elucidate insulin’s influence on neuroinflammation and neurodegeneration [8]. Recent studies corroborate the findings of You et al. regarding insulin’s influence on microglial activation in Alzheimer’s disease (AD) pathology. A study published in *Neurochemistry International* emphasized that dysfunctional insulin signaling contributes to neurodegeneration and AD. The research highlighted that insulin resistance leads to neuroinflammation, with microglial activation playing a pivotal role in disease progression. Key molecules such as IRS, PI3K, Akt, and GSK-3β are implicated in these processes, suggesting that impaired insulin pathways exacerbate neurodegenerative conditions [9]. Collectively, these studies underscore the critical role of insulin in regulating microglial activity and its broader impact on neuroinflammation and neurodegeneration in Alzheimer’s disease.

## 9. Microbial Infections and AD

The association between microorganisms and AD pathogenesis is an emerging field of study. Yadav et al. reviewed fungal and bacterial infections in AD brains, revealing interesting connections. The presence of polymicrobial infections may contribute to uncontrolled neuroinflammation and neurodegeneration, influencing disease progression. The manuscript underscored the importance of investigating microbial involvement in AD etiology for targeted anti-inflammatory therapeutic approaches [8]. Recent studies [10,11] support the association between microbial infections and Alzheimer’s disease (AD) pathogenesis, as highlighted by Yadav et al. Collectively, these studies underscore the significance of investigating microbial involvement in AD etiology. They highlight the potential of targeted anti-inflammatory therapies to mitigate neuroinflammation and slow disease progression.

## 10. Ubiquitin as a Biomarker for Early Cognitive Decline

Lastly, the potential of ubiquitin as a blood biomarker for early cognitive decline was assessed by McFarlane et al. The study analyzed plasma ubiquitin levels in individuals with different cognitive functioning levels. While no significant disparities in ubiquitin levels were identified, the study emphasized the need for further research to evaluate ubiquitin’s potential as a neurodegeneration biomarker [12]. Conversely, research by Hattori et al. explored cerebrospinal fluid (CSF) ubiquitin levels in patients with neurodegenerative diseases. They reported that increased CSF ubiquitin concentrations correlated with disease severity, indicating potential as a biomarker in neurodegenerative conditions [13]. These findings suggest that while plasma ubiquitin levels may not be indicative of early cognitive decline, CSF ubiquitin concentrations could have potential as biomarkers in neurodegenerative diseases. Further research is necessary to clarify ubiquitin’s role and efficacy as a biomarker in different biological fluids.

## 11. Conclusions

The array of findings presented in these nine manuscripts adds valuable pieces to the complex puzzle of Alzheimer’s disease. From novel drug candidates to genetic associations, microbial involvement, and biomarker exploration, each study contributes to our current knowledge of AD’s multifaceted pathogenesis. Collectively, these insights pave the way for innovative therapeutic strategies and personalized approaches to combat this devastating neurodegenerative disorder. Collaboration among researchers in diverse fields will be crucial to unraveling the intricate mechanisms underpinning AD and devising effective interventions to improve the lives of those affected by this condition.
